# Medical Opinion and Movement

**Published:** 1908-06-06

**Authors:** 


### Medical Opinion and Movement.

Hirschfeld, in a clinical study of several cases of pernicious anæmia, expresses the opinion that the prognosis in this disease is not always so gloomy as it is usually considered. Under proper treatment, it is possible to keep the patients alive and active for many years, and the author records cases in which the patients were following their ordinary business six years after they had been treated. The general conclusion drawn from this study, is, perhaps, much too optimistic, and the question naturally arises, whether all these patients suffered from typical pernicious anæmia, or whether, as seems more probable, they were really cases of so-called pseudo-pernicious anæmia, that is to say, of a severe anæmia with hæmolytic changes. The author admits that in every case there can be no question, as yet, of a complete cure. The patients are always liable to a "recurrence" which may "occur with extraordinary rapidity." The author records one interesting case, which shows how sudden and how severe such relapses may be. A patient who, two years before, had been treated with success for pernicious anæmia, came to the clinic declaring that he was again suffering from the disease. The blood examination was, however, entirely negative, the patient was reassured and sent home with instructions to report himself again if he felt worse. Ten days later he returned, very much worse in appearance and general health, and a blood examination now gave the typical "pernicious anæmia" result. The author, therefore, pleads for monthly blood examinations as a routine practice in every case, whether the patient feels well or not. Such a blood examination is of great value from a prognostic point of view, but too much weight ought not to be laid upon it to the exclusion of other considerations, as the blood condition itself is rarely an index of the general state of the patient. Much more important, therefore, is the methodical investigation of the several systems, especially of the lungs and heart.

At the recent German Surgical Congress in Berlin, Professor Bier, who is at the head of the Royal Surgical Clinic at Berlin, and who is best known to English readers through his introduction of the hyperæmic method of treatment, demonstrated a new method of local anæsthesia, by direct injection of the solution into the superficial veins. The principle of the new method is to make the anæsthetic solution act upon the peripheral nerves as quickly as possible, and this, according to Bier, is best done by injecting the solution directly into a vein, after the part has been made bloodless by a previous application of the Sylvestri-Esmarch bandage. The technique of the method is simple, and can be easily mastered, but exactness and care are essential to success. Carried out in accordance with the principles laid down by Bier, it is a method which appears to be especially suited to operations on the extremities. Thus, in a case of resection of the knee for old tubercular ankylosis, the patient's leg was elevated and carefully rendered bloodless by means of the bandage. A small amount of novocaine solution was then injected at the site of the incision, over the internal saphenous vein, the vessel chosen for the injection. The vein was found and opened, and an ordinary infusion cannula, attached to an injection syringe, introduced into its lumen, and 150 c.c. of a ½ per cent. novocaine solution injected. Anæsthesia was complete from the thigh downwards after fifteen minutes, and the operation was proceeded with. Finally—a point on which Bier lays stress—the solution is washed out of the vein by a stream of normal saline, and the small wound closed. The method, however undesirable it may sound in theory, has been found to be excellent in practice and is now regularly used in the clinic, and it is one which deserves to be more widely known.

The latest statistical returns for the German Empire, show clearly how the number of suicides in Germany has increased of late. At one time, Germany stood comparatively low down in the list of "suicidal countries," and a long way behind France; at present it possibly heads the list. Thus, in 1906, 12,495 suicides were registered, of whom 2,922 were females. This works out at the high figure of 41.2 per 100,000 of population. The northern provinces show the smallest number; the large commercial centres, such as Bremen, Berlin, and Hamburg, head the list. No statistics are given from which it is possible to draw conclusions as to the probable factors which are responsible for this high figure, but from time to time attention is drawn to the fact that a large number of these cases of suicide are drawn from the unemployed and from the poorer classes, and that the percentage of juvenile suicides is relatively much higher in Germany than in France.

Ricci, in the *Gaz. Med. di Roma*, gives an account of his investigations into the therapeutic value of grape-ferment in the treatment of diabetes. As the result of observations upon six patients suffering from diabetes, and treated by strong doses of ferment over a long period of time, he is led to think that the ferment has a marked effect in reducing glycosuria and acidosis, as also in diminishing such symptoms of the disease as polyuria, visual troubles, pruritus, articular pains, etc. Even when the disease is complicated by an associated albuminuria, he claims that the curative effect of the ferment is well marked, the albuminuria disappearing along with the glycosuria. Owing to the fact that the condition of the patient remains good for some time after he has ceased to take the ferment, Ricci is led to believe that the action of the ferment is cumulative. He recommends that large doses should be given and continued for long periods of time, but as gastrointestinal disturbances are likely to occur at the outset of the treatment, he advises small and gradually increasing doses until such time as toleration shall have been acquired.

DR. N. J. BLACKWOOD, of the United States Navy, reports in the *Annals of Surgery* an extraordinary case of fracture of the atlas and axis vertebræ, with forward dislocation of the skull on the spinal column, in which, by artificial respiration, life was preserved for thirty-four hours. Artificial respiration was commenced at once after the gymnasium accident which caused the lesion; the patient never lost consciousness and could reply to questions by nodding and winking, and even, when aided by extra forcible compression of the chest in expiration, by a few spoken words. There was no organ below the larynx which performed its function, the heart alone excepted. The patient suffered from slight pain in the head, but was elsewhere completely anæsthetic, so much so, that a laminectomy was performed without causing any pain. Unfortunately, the cord was completely pulped as it issued from the foramen magnum, and so the operation was of no avail. Great difficulty was experienced in its performance, for owing to the necessity of maintaining artificial respiration, the surgeon had to sit on a low stool and operate in the manner of a fresco painter engaged on a ceiling. The case is reported as unique in duration, for the only other record of similar injuries, not immediately fatal, deals with one in which life was prolonged only three and a half hours.

IN the same issue, Dr. J. M. Mason, of Birmingham, Alabama, analyses the results of treatment in all published cases of fracture-dislocation of the upper end of the humerus since 1894, including dislocation with fracture of the great tuberosity. The number of cases is eighty-four, a number large enough to yield valuable inferences as to the results of modern surgical treatment in this grave injury. As a result of his researches, he concludes that excision should only be practised when reduction by open arthrotomy has failed, or in fractures of the anatomical neck, when the condition of the fragment does not justify the expectation of union. The after results of reduction are in many cases extremely good, for fourteen out of twenty-three patients recovered without any impairment of function, and others did fairly well. In fracture of the great tuberosity, it is advised to nail the fragment into position, after reduction of the head, in recent cases; and to remove it if it causes impaired function in old ones. It is especially urged that operative measures should be adopted without delay, whenever manipulation fails to secure reduction, and these may include screwing or wiring of the fracture, as well as the treatment of the dislocation. Excision, however, often gives very fairly good results, and although, as a rule, inferior to open arthrotomy, reduction, and treatment of the fracture, it is to be preferred to ankylosis with excessive callus around badly displaced fragments. It is also often necessary in long standing, or neglected cases. The value of *x*-ray examination is also greatly emphasised in this exhaustive paper; and if any proof of this were wanting, it might be supplied by pointing out that before the application of that discovery to surgery, fracture of the great tuberosity was considered a curiosity, whereas it is now known to be by no means uncommon.

DR. G. F. SHIELS, of New York, raises the interesting question as to what is the origin and nature of colic. He has consulted medical literature on the subject, and has addressed the question to a large number of well-known teachers of medicine and surgery, and as a result he draws up the following epitomised description of colic:—Colic is an acute, violent, paroxysmal, spasmodic, periodic, gripy pain due to contraction of a hollow viscus or structure, whose walls are composed of smooth or non-striated muscle, with irritation of the sensory nerves therein, and is produced by an effort on the part of such viscus or structure to rid itself of an irritant or obstruction. It has been described as a pain wave which has a gradual period of onset, lasting seconds or minutes, a climax and a gradual subsidence. It may be due to a reflex from other organs than the one affected, and also to certain poisons such as lead. Dr. Max Einhorn briefly defines it thus: "Under the name of colic we usually understand painful spasmodic contraction of the non-striated muscle of any viscus." It will certainly be readily admitted that these definitions conform to the generally accepted notion of the nature of colic, and that one associates it especially with excessive contractions of non-striated muscle. Dr. Shiels, however, joins issue with this description, and his views are certainly worthy of consideration.

DR. SHIELS contends that this description lacks an essential element—that *without the presence of peritoneum there can be no colic.* He defines colic as an acute spasmodic *peritoneal pain wave* generally associated with an effort of a hollow viscus with walls of non-striated muscle, to rid itself of an irritant or obstruction, such viscus being either *covered by or in close relationship to the peritoneum.* In support of this theory of the peritoneal origin of colic he points out that colic is only associated with structures covered by peritoneum. Blood-vessels, lymphatics, the vas deferens, the urethra, the Eustachian tubes, ducts of certain glands, the trachea and bronchi, the œsophagus, the rectum—all have walls with non-striated muscular fibres, and are supplied with afferent and efferent nerves. When obstructed or irritated they give rise to a sense of pain, but in no instance is this pain colic. Dr. Shiels repudiates the idea that visceral peritoneum is insensitive, and considers that the abdominal sympathetic as well as the vagi, phrenic and cerebrospinal nerves are collectively the tracts through which this especial pain sense is transmitted, and the fact of their being conducted and supported towards their destination by means of the peritoneum probably alters the character of the pain waves and gives it that peculiar type which we understand by the term colic. The multitudinous connections and ganglionic anastomoses of these nerves explain the violent and peculiar symptoms brought about by a relatively trivial excitation, such as a plug of mucus in the appendix. In this way also the extensive associated reflex disturbances so often found in conjunction with severe colic may be accounted for: disturbances of the vasomotor system, evidenced by sweating and pallor; cardiac disturbances; disturbance of the vomiting centre with nausea and vomiting; renal anuria; and so forth. The diffuse radiating character of colic, often misleading in diagnosis, is also urged as a point in favour of this peritoneal theory. Dr. Shiels records a case in which an aseptic gauze sponge left in the peritoneal cavity, and found subsequently wrapped within the greater omentum, not touching either bowel or parietal peritoneum, gave rise to the most violent colic he has ever witnessed.

---

The administration of a hypodermic injection of morphia previous to general anæsthesia has often been advocated, and at least in certain cases is found a useful practice. Dr. Strauch, however, advocates a very free use of sedatives previous to operation. On the night previous to operation he gives an adult patient fifteen grains of veronal. One hour before operation he gives a hypodermic injection of about a quarter of a grain of morphia, and at the same time a rectal injection of alcohol with 5 to 10 minims of tincture of opium and a pinch of salt. The veronal secures to the patient a good sleep instead of a restless night with the torments of anxious anticipation; and he finds that it is free from the usual depressing cardiac effects of other hypnotics. By these measures the patient is often asleep when the anæsthesia is commenced, and the patient is brought into the theatre in bed to avoid awakening him. Ether is given by the open method with a mask, and anæsthesia is rapidly obtained. There is no excitation stage even in alcoholic patients, and less anæsthetic is required to maintain a satisfactory depth of unconsciousness. The veronal causes further sleep for some hours after the operation, and vomiting is generally absent. From the patient's point of view this method of preparation must be extremely pleasant; there is, however, reason to doubt the desirability of submitting him to the influences of such powerful drugs at a time when his physical strength may be heavily taxed by a severe operation. Fifteen grains of veronal seems a large dose. Ten grains are generally sufficient to secure several hours' sleep, and although it is undoubtedly a very satisfactory hypnotic, it is not wholly free from the depressing effects which invariably accompany the use of any hypnotic hitherto discovered.

---

So much has been written and taught as to the danger of administering morphia to infants and young children that it is interesting to note some conditions in which the use of the drug has led to good results. Ausset, in *L'Echo Médicale du Nord* describes some cases of spasmodic and diphtheritic laryngitis which came under his care after his attention had been directed to the use of morphia in such cases by Lesage. He treated these by the injection hypodermically of half a centigramme of hydrochlorate of morphia. In all cases he was able to control laryngeal spasm by the drug, and was in no case forced to resort to tracheotomy or intubation to relieve the dyspnœa. His results persuade him that the drug should be employed in cases of diphtheria with spasm of the glottis, even when diphtheria-antitoxin is at hand, and always before recourse is had to tracheotomy or intubation. The alkaloid alone should be used. Ausset attributes the bad results obtained by some observers to the use of other preparations of opium. Although he has in many cases tried codeine, he finds its use fraught with much less certain results, and has therefore discarded it in favour of morphia. He advocates the use of the relatively large dose of half a centigramme, and finds the effects thereof to last from six to eight hours. He has experienced no injurious after-effects. The communication is of interest, as it may fall to the lot of any practitioner to be called upon, in a hurry, to treat a case of severe dyspnœa due to laryngeal spasm. The necessary intubation or tracheotomy apparatus may not be handy, and tracheotomy is not an easy operation in infants under the best conditions. A hypodermic needle and syringe are almost always at hand, and an injection of morphia would seem to be at least justifiable under these conditions. It would be interesting to learn whether the experience of other observers with morphia in such cases confirms the results of Ausset and Lesage.

---

Mr. Macleod Yearsley summarises in the *British Journal of Children's Diseases* the aural manifestations of inherited syphilis. Middle-ear inflammations in these children do not differ from those in others, but specific treatment must be carried out. It is the labyrinthine affections that are the most characteristic and important. There are two separate varieties pathologically, and they are recognisable clinically by the presence or absence of vertigo. The latter class are the most numerous, and in the patients other stigmata of syphilis are practically always present. The symptoms are gradually increasing deafness, commonly in adolescents and young adults, and the associated lesion is chronic specific osteitis of the labyrinth leading to complete occlusion. In the cases with vertigo the symptoms are due to increased tension caused by exudation, and the age of the patients may be anything up to fifty years. Although a certain number of ears are destroyed by syphilis before or within a few months after birth, the number of deaf mutes who have this disease is relatively very small, as most of the sufferers are over eight years old when attacked. It is a curious observation that in many cases the deafness has become first evident during a course of treatment for interstitial keratitis or other inherited lesion, and Mr. Yearsley also notes that active mercurial treatment for the deafness has very little effect. Pilocarpine has given good results in three cases in which he has tried it, but it must be given early to be of any benefit. The true *rôle* of mercury is as a prophylactic in infancy, for it is very seldom that ear symptoms are found in any but those in whom this has been neglected.

